# 

**Published:** 1844-01

**Authors:** 


### CONTENTS

OF NO. I.

---

### ORIGINAL COMMUNICATIONS.

ESSAYS AND CASES.

Art. I.—Remarks upon Inversion of the Uterus. By W. L. Sutton, M.D., of Georgetown, Ky. - - 1
Art. II.—A Case of Cataract Complicated with Tremulous Iris, successfully treated. By Wm. M. Boling, M.D., of Montgomery, Ala. - - - - - 17
Art. III.—Complete Aphonia, successfully treated. By Martial Dupierris, M.D., of Havana. - - 19
Art. IV.—Remarks on the Cure of Consumption. By Wm. A. M'Dowell, M.D., of Louisville, Ky. - - 21

REVIEWS.

Art. V.—The Elements of Materia Medica and Therapeutics. By Jonathan Pereira, M. D., F. R. S. & L. S., &c. With numerous illustrations. From the Second London Edition, enlarged and improved. With notes and additions. By Joseph Carson, M.D., Professor of Materia Medica, &c. In 2 vols., 8vo. pp. 714 and 852. Philadelphia. Lea & Blanchard. 1843. 24
Art. VI.—Practical Manual of the Diseases of the Heart and Great Vessels. A work intended to facilitate and extend the study of these Diseases. By F. A. Aran, Interne of the First Class of the Hotel-Dieu, &c. Translated from the French; by Wm. A. Harris, M.D. Philadelphia. Ed. Barrington & Geo. D. Haswell. 1843. pp. 296, 12mo. - - - - - 46
Art. VII.—A Treatise on Dislocations of the Joints. By Sir Astley Cooper, Bart. F. R. S., &c. A new edition, much enlarged. Edited by Bransby B. Cooper, F. R. S., &c. With additional observations, and a memoir of the author. Piladelphia: Lea & Blanchard. 1844. pp. 499. 8vo. - - - - - 72

SELECTIONS FROM AMERICAN AND FOREIGN JOURNALS.

On the successful employment of Belladonna as a prophylactic during the prevalence of Epidemic Scarlatina, - 75
Seats of Tubercle. - - - - - 75
Remedy for Cough. - - - - - 76
An Elegant Sticking Plaster. - - - - 76
On the Use and Abuse of Filing to remove Irregularities of the Teeth. - - - - - - 77
Œdopsophia, or Noisy Expulsion of Gas from the Vagina. 78
French Gratitude to Medical Men. - - - 79
Dr. Sibson on the Means of Determining the Relative Position of the Thoracic and Abdominal Viscera. - - 80

ORIGINAL INTELLIGENCE.

Western Journal, - - - - - - 82
The Natural Means of Preserving Health, - - 83
Euphorbia Corollata, - - - - - - 86
Kentucky Lunatic Asylum, - - - - - 87
Professor Bache's Introductory, - - 90
Anatomical Atlas, - - - - - - 91
Wilson's Dissector, Books received, Western Medical Schools, 92

